# Accuracy of Opportunistic Bone Mineral Density Assessment on Staging Computed Tomography for Gynaecological Cancers

**DOI:** 10.3390/medicina57121386

**Published:** 2021-12-20

**Authors:** Catherine Anne O’Gorman, Sarah Milne, Gerard Lambe, Aleksandra Sobota, Peter Beddy, Noreen Gleeson

**Affiliations:** 1 Trinity College Dublin, School of Medicine, Department of Obstetrics and Gynaecology, D02 PN40 Dublin, Ireland; noreengleeson@dubgyn.org; 2 Department of Gynaecological Oncology, St James’s Hospital, D08 NHY1 Dublin, Ireland; sarah.milne@ucdconnect.ie (S.M.); sobota.ola@gmail.com (A.S.); 3 Department of Radiology, St James’s Hospital, D08 NHY1 Dublin, Ireland; Gerard.lamb@ucdconnect.ie (G.L.); pbeddy@stjames.ie (P.B.)

**Keywords:** oncology, bone health, survivorship, care-gap, osteoporosis

## Abstract

*Background and Objectives*: Women with gynecological cancers constitute a high-risk cohort for loss of bone density. International guidance stipulates women undergoing cancer treatments associated with bone loss should have a quantitative assessment of bone density. Access to Dual-energy X-ray Absorptiometry (DXA) is limited. This study aimed to assess the accuracy of opportunistic bone density measurement on staging computed tomography (CT) scans for gynaecological malignancies, in comparison to the gold standard DXA. *Materials and Methods*: Women with a staging CT scan of the abdomen and pelvis for a new diagnosis of gynecological cancer were recruited. DXA was performed within 6 weeks of treatment for gynaecological cancer. Lumbar bone density was measured by CT attenuation values, in Hounsfield units (HU), of the anterior trabecular region. Correlations between CT and DXA parameters were analysed. Receiver Operating Characteristic(ROC) curves for diagnosis of low bone density and osteoporosis were analysed. *Results:* Final cohort included 48 of 50 women recruited. There was good diagnostic accuracy for abnormal bone density and osteoporosis, with areas under the ROC curve at L1 of 0.77 (*p* = 0.002) and 0.80 (*p* = 0.020) respectively. CT-HU of 170–190 yielded sensitivities of 87–90%, positive predictive values of 75–84% and negative predictive values of 71–75% for the diagnosis of low bone mineral density. CT-HU of 90–110 yielded specificities of 85–93% for the diagnosis of osteoporosis. Moderate correlations were found between CT-HU and both DXA T-scores and diagnostic categories. *Conclusions:* This is the first study to assess the opportunistic application of CT in the assessment of bone health in women with gynaecological cancer, a cohort at high-risk of osteoporosis. The correlation between bone density assessment in CT-HU and DXA, and strong AUC values for the diagnosis of low bone density (0.77) and osteoporosis (0.80) support this pragmatic solution in resolving the care-gap in cancer treatment-induced bone loss, often associated with poor access to DXA.

## 1. Introduction

The UK and Ireland are categorised as high risk for osteoporosis, with an estimated 21.8% of women over 50 years having osteoporosis, 35–50% of women aged over 50 years will sustain an osteoporotic fracture and 20% will suffer a hip fracture in their lifetime. Northern Europe has higher rates again with Swedish women over 50 years holding a 23% lifetime risk of hip fracture [1,2,3,4]. Women with gynecological cancers are at increased risk of developing osteoporosis through cancer treatment induced bone loss (CTIBL) which is associated with increased all-cause mortality [5,6]. Iatrogenic hypo-estrogenism (particularly early menopause due to surgery, chemotherapy, or radiotherapy), and the direct effects on bone density of pelvic radiation result in an accelerated loss of bone mineral density (BMD) [7,8,9,10]. Pelvic insufficiency fractures are common following external beam radiation therapy for gynecological cancers [11]. International guidance stipulates women commencing CTIBL-associated therapies should have quantitative BMD assessment at baseline [10,12]. The gold standard for quantitative BMD assessment is Dual-energy X-ray Absorptiometry (DXA) [13]. Additionally advised, are FRAX fracture probability calculations, calcium and vitamin D assays, consideration of supplementation, and based on DXA and clinical risk factors, the commencement of antiresorptive medications for those at high risk.

Randomised controlled trials of screening, consisting of DXA assessment following identification of high FRAX fracture-probability scores, demonstrated a substantial reduction in major osteoporotic fractures, most benefiting those at highest risk [14,15]. Access to DXA is very varied regionally and socio-economically. Previous research at our cancer unit demonstrated a care-gap in the attention to bone health of women with gynecological cancers, with low rates of referral for quantitative BMD assessment [16]. We considered that limited access to DXA might be contributing to this deficiency and looked at other facilities for measuring BMD.

The substantial radiation exposure with CT makes it unsuitable for routine measurement of bone density but the majority of women diagnosed with gynecological cancers already undergo CT abdomen and pelvis (AP) for cancer staging and treatment planning. The opportunistic use of CT to assess BMD in a high-risk population is attractive for a number of reasons. Consideration of patients’ time, satisfaction and radiation exposure coupled with healthcare economics, dictates that information obtained from each radiological investigation ought to be optimised when clinically appropriate. Opportunistic quantitative assessment of bone density in CTs performed for other indications has been reported previously, but not in the field of gynaecologic oncology [17,18,19,20]. The aim of this study was to assess the accuracy of opportunistic assessment of lumbar vertebral BMD on staging CTs for gynecological malignancies, in comparison to the current gold standard diagnostic for osteoporosis, DXA.

## 2. Materials and Methods

### 2.1. Study Design

This was a prospective, cross-sectional diagnostic accuracy study of bone density as measured by computed tomography (CT) compared to the gold standard DXA, in a pilot introduction of opportunistic bone densitometry using CT in a gynecological oncology service. Densitometric measurements of trabecular bone from CT were correlated with areal BMD measurements, T scores and diagnostic categories determined by DXA. The study cohort consisted of women undergoing CT at initial diagnosis of gynecological malignancy, and prior to any treatment-effect on BMD. These women were identified from sequential referrals to the gynaecologic oncology department within a six-month period (March–August 2019). Inclusion criteria were women, aged over 18 years presenting with gynecological cancer, undergoing CT AP for cancer staging. Exclusion criteria included lack of capacity to consent, and clinical condition precluding transfer for DXA. Women who met the inclusion criteria were offered a DXA scan, to be performed before or within six weeks of commencing treatment for gynecological cancer. The six-week limit was chosen to facilitate both inclusions in the study and avoid interruption to the prompt commencement of treatment. Each participant received written information and met with a member of the research team to discuss the study, prior to obtaining informed, written consent to participate. Patient demographic and treatment information was prospectively collected, anonymised, and stored for analysis.

### 2.2. Radiological Methodology

Central DXA was performed on a single GE Lunar Prodigy Advance by a single radiographer. Areal BMD was calculated in g/cm^2^ for hips and lumbar vertebrae L1–L4. T-scores were calculated for hips, L1 and lumbar mean, using the ISCD and manufacturer recommended standards. The lowest single-site T-score (hip or lumbar spine) was used to allocate each participant to the diagnostic categories of osteoporosis, osteopaenia or normal BMD.

CT AP imaging was performed in our unit’s radiology department or accepted from referring institutions. This was to optimise the evaluation of a real-world approach. Kilovoltage was standardised to 120 kV. CT images were analysed by a specialist registrar in radiology (GL), under the supervision of a consultant radiologist (PB), both blinded to patient demographics and DXA results. Measurement of lumbar vertebral CT attenuation was performed at a standard PACS workstation with the use of the bone window setting [21]. A single ovoid region-of-interest (ROI) is placed in the trabecular region of each anterior vertebral body (T12-L5) in an axial plane, with avoidance of focal bone lesions, such as fractures [17]. The mean attenuation of this ROI, in Hounsfield Units (HU) represents bone density. The presence of vertebral compression fractures was assessed on sagittal spine reformats using the Genant semi-quantitative method, with recording only of moderate and severe deformities to minimise ambiguity [22]. This simple ROI technique has been shown to compare favourably with more complex quantitative CT techniques for measuring BMD. It is faster to perform, does not require dedicated software, has minimal interobserver variability, and is unaffected by intravenous contrast [19].

### 2.3. Statistical Analyses

Participant demographics and clinical characteristics are described. Correlation between the CT BMD (HU) and DXA assessment of BMD (areal and T-score) was investigated by calculation of Pearson correlation coefficient. Calculation of Spearman’s rho was performed to assess the correlation between the CT BMD and DXA diagnostic categories (Normal, Osteopaenia, Osteoporosis). The one-way ANOVA was used to investigate the difference between the mean CT BMD of each of the DXA-designated diagnostic categories. This was performed for each vertebral level. Receiver operating characteristic curves were constructed for the differentiation of normal from reduced bone density, and osteoporosis from osteopaenia/normal bone density. The area under the curve (AUC) was calculated in each instance. Threshold analysis was undertaken to calculate sensitivity, specificity, positive and negative predictive values for each potential threshold. Youden’s J statistic was calculated to identify the statistically optimal threshold values. Statistical analyses were carried out using SPSS software and statistical significance was designated to *p* < 0.05.

## 3. Results

### 3.1. Cohort Demographics

The study cohort consisted of fifty sequential women with newly diagnosed gynecological cancer and CT staging. The median time-interval between CT and DXA was 31.5 days. Two participants had CTs performed at 100 kV and were excluded from the final analysis. The patient demographics and clinical risk factors are summarised in Table 1. The median age at diagnosis was 59 (range 31–86). Cancer treatments included surgery (*n* = 45, 90%), external beam radiotherapy (*n* = 21, 42%) and chemotherapy (*n* = 18, 36%). Thirteen (26%) women were pre-menopausal at diagnosis and 92% underwent iatrogenic menopause. Two(15%) premenopausal women had pelvic radiotherapy.

Table 1 summarises participant demographics and clinical risk factors.

Patients were categorised according to the WHO diagnostic criteria for DXA assessment of BMD into three groups: osteoporosis (T-score ≤ −2.5) was diagnosed in seven women(14%), osteopaenia (T-score ≤ −1.0) in 26 (52%), and normal BMD in 17 (34%) women. The mean areal BMD, DXA T-score, and CT-HU values for each DXA derived diagnostic category are summarised in Table 2. The mean areal BMD and CT-HU for each vertebral level are summarised in Appendix A Table A1.

The mean areal BMD, DXA T-score, and CT-HU values for each DXA derived diagnostic category are summarised in Table 2.

### 3.2. Correlation between CT and DXA in Measurement of Bone Mineral Density

**Figure 1:**
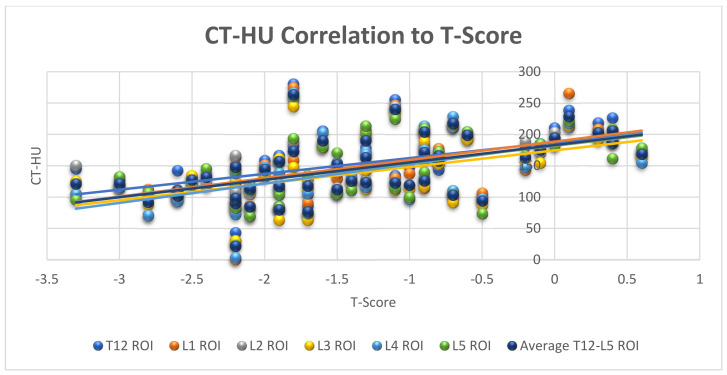
Moderate correlation between CT assessment of bone density (CT-HU) at each vertebral level and DXA T-score. The mean Pearson’s correlation coefficient was 0.53, (range 0.50–0.56, *p* = 0.000).

The relation between CT-HU and DXA-derived T-score is shown in Figure 1. There was a correlation, of statistically moderate strength, between CT-HU (each level) and DXA T-score with mean Pearson’s correlation coefficient of 0.53, (range 0.50–0.56, *p* = 0.000) for each vertebral level. There was a correlation of statistically moderate strength between the CT-HU and the diagnostic categories derived from DXA assessment with Spearman’s rho mean value 0.49 (range 0.43–0.56, *p* < 0.005 for all levels). The correlation between the CT-HU and the areal BMD as determined by DXA was assessed and yielded a statistically significant correlation, of moderate strength for L1 and L3 levels (Pearson’s correlation coefficient of 0.40 and 0.31 (*p* = 0.005 and 0.037) respectively). There was a trend towards significance for L2 with a Pearson’s correlation coefficient of 0.29 (*p* = 0.056) and no significant correlation was found for L4 (Pearson’s correlation coefficient of 0.23, *p* = 0.175).

**Figure 2:**
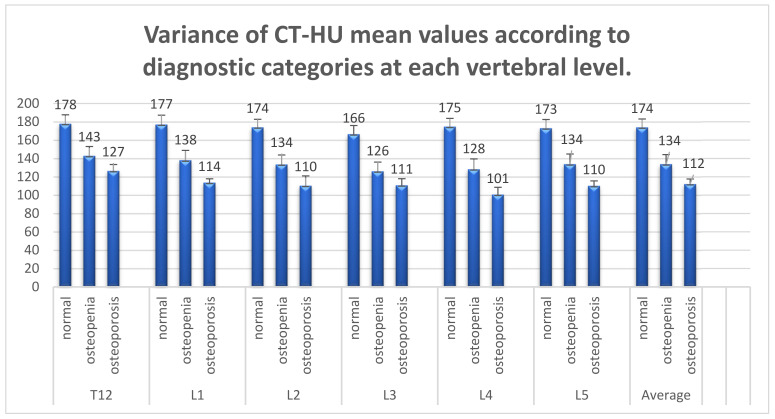
Variance in the mean CT-HU according to DXA diagnostic categories at each vertebral level.

A statistically significant difference was demonstrated between the mean CT-HU in each DXA diagnostic category, for each of the vertebral levels (e.g., L1: (F(2,45) = 5.415, *p* = 0.008). The results of the ANOVA and posthoc analyses are shown in Table A2. The variance in the mean CT-HU according to DXA diagnostic categories at each vertebral level is summarised in Figure 2. Post-hoc analyses showed statistically significant differences between the normal BMD group and both the osteoporosis and osteopaenia groups. No statistically significant difference was shown between the osteoporosis and osteopaenia groups.

### 3.3. Analysis of CT Diagnostic Capabilities in Bone Densitometry

**Figure 3:**
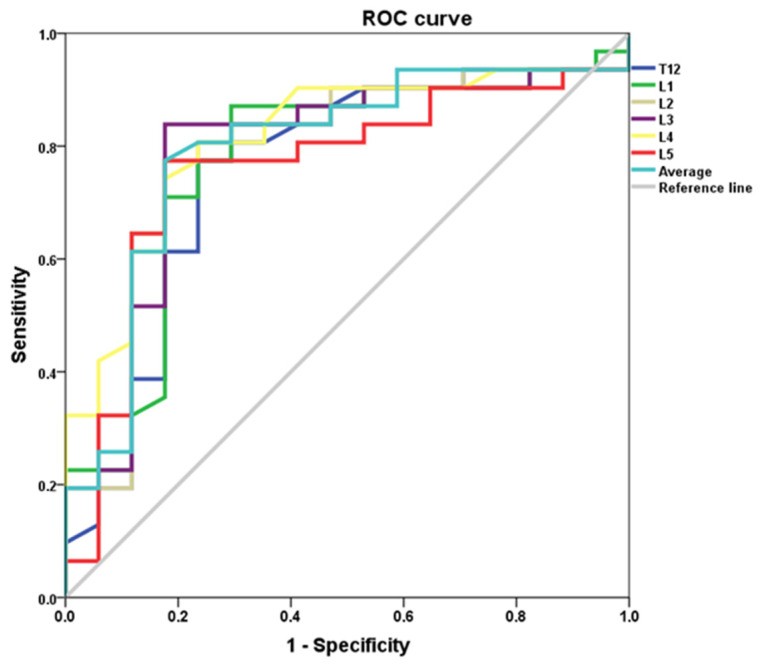
ROC curve for CT diagnosis of abnormally low bone density (osteopaenia and osteoporosis).

The diagnostic capability of CT in detecting below normal BMD (T ≤ −1.0), was assessed using receiver operating characteristic (ROC) curve analysis and calculation of the area under the curve (AUC). The AUC for differentiating low BMD from normal BMD at the L1 vertebral level was 0.77 (*p*= 0.002, 95% CI 0.62–0.92). The AUC results for diagnosing abnormally low BMD for the remaining vertebral levels and the vertebral average are shown in Table 3. The ROC curves for each level are represented in Figure 3.

The AUC results for diagnosing abnormally low BMD and osteoporosis for each vertebral level and the vertebral average are shown in Table 3.

The diagnostic capability of CT in detecting below normal BMD (T ≤ −1.0) and osteoporotic (T ≤ −2.5), was assessed using receiver operating characteristic (ROC) curve analysis and calculation of the area under the curve(AUC). Figure 3 depicts the ROC curves for diagnosing below normal bone mineral density at each of the vertebral levels.

**Figure 4:**
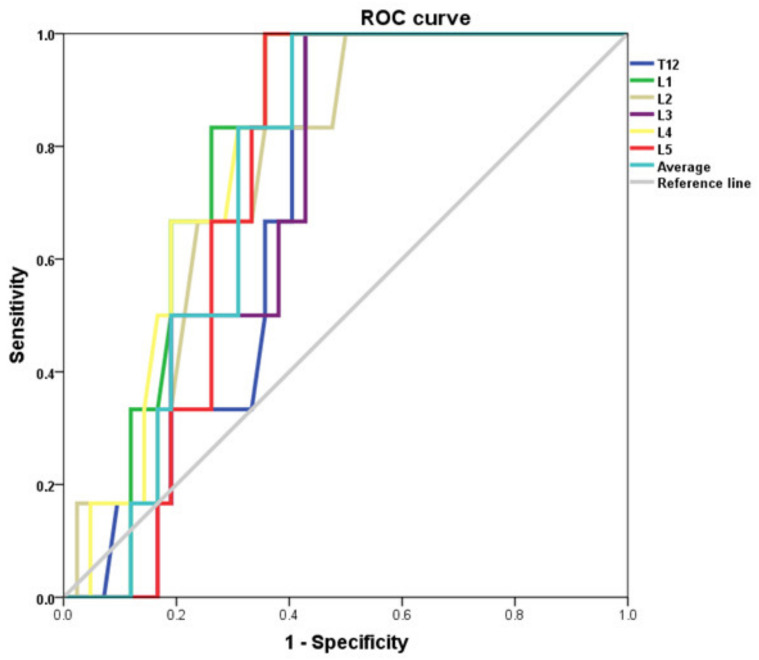
ROC curve for CT diagnosis of osteoporosis (DXA T-score ≤ −2.5).

ROC analyses were performed to assess the ability of CT to diagnose osteoporosis. For this analysis, the AUC for the L1 level was 0.80 (*p* = 0.02, 95% CI 0.67–0.92). The AUC values for the remaining vertebral levels and the vertebral average are outlined in Table 3 and represented in the ROC curves in Figure 4.

The diagnostic capability of CT in detecting below normal BMD (T ≤ −1.0) and osteoporotic (T ≤ −2.5), was assessed using receiver operating characteristic (ROC) curve analysis and calculation of the area under the curve(AUC). Figure 4 depicts the ROC curves for diagnosing osteoporotic bone mineral density (T ≤ −2.5) at each of the vertebral levels.

Threshold analysis, calculating the sensitivity, specificity, positive predictive value (PPV), and negative predictive value (NPV) for a range of thresholds at the L1 vertebral level was performed. Youdon’s J statistic was also calculated to identify the statistically optimal CT-HU cut-off at each vertebral level for diagnosis of low and osteoporotic BMD. A selection of the most clinically useful thresholds for application of CT-based BMD assessment, and associated sensitivity and specificity values, is presented in Table 4 while a broader record of cut-offs and their sensitivities and specificities for L1 vertebral level is found in Table A3. A complete record of all thresholds with diagnostic performance for each vertebral level is found in the supporting information accompanying this article (Supplementary material, Tables S1 and S2).

A selection of the most clinically useful thresholds for application of CT-based BMD assessment, and associated sensitivity and specificity, positive and negative predictive values is presented in Table 4. The statistically optimal diagnostic thresholds for the L1 vertebral level as identified by the Youdon index are also shown.

## 4. Discussion

Osteoporosis is underdiagnosed in women with gynecological malignancies. This may be due, in part, to limited access to DXA. The primary aim of this study was to assess the accuracy of BMD assessment on staging CT scans. This is the first study examining this opportunistic application of CT in gynecological oncology. The patient demographic, with median age 59, higher BMI, the predominance of endometrial cancer and three-quarters postmenopausal at diagnosis is representative of our gynecological cancer population.

Just over one-third (34%) of women had normal BMD, over half (52%) had osteopaenia, and almost one in six (14%) had osteoporosis. One woman had four vertebral compression fractures. That two-thirds of women had impaired bone density before commencing cancer treatment is evidence of the need to challenge this aspect of women’s health as part of the best cancer survivorship practice. We emphasise that all women with gynecological cancer merit full BMD screening.

Moderate correlations were found between CT-HU and DXA T-scores and diagnostic categories at each vertebral level. A statistically significant difference emerged in mean CT-HU between the normal BMD group (174 HU) and both the osteopaenia (134 HU) and osteoporosis (112 HU) groups. The difference between the osteoporosis and osteopaenia groups was not significant and we acknowledge the small osteoporosis sample size.

This study demonstrates good diagnostic accuracy for CT for abnormal BMD and osteoporosis, AUCs at L1 of 0.77 (*p* = 0.002) and 0.80 (*p* = 0.020) respectively. Similar AUCs (0.74–0.83) are described in non-gynecological, larger series of opportunistic CT assessment of BMD [19,23].

Following previous research identifying a care-gap in attention to bone health in gynecological oncology, this study identifies a potential pragmatic solution in the use of opportunistic BMD assessment at routine staging CT [16]. We employed established BMD assessment techniques in a study design designed to be pragmatic with respect to real world application. We accepted CTs performed at referring institutions to widen applicability while controlling for kilovoltage.

The sample size is modest and a small number of participants met the DXA criteria for osteoporosis. The lack of a statistically significant difference between the osteoporosis and osteopaenia groups may be attributable to that small osteoporosis cohort. The cross-sectional nature of the study precluded the use of clinically measurable future outcomes like fractures.

We applied the Youdon index to identify statistically optimal thresholds but acknowledge that this cannot be expected to provide the most clinically relevant thresholds. The determination of clinically relevant thresholds is underpinned by the proposed clinical application. If identifying patients unlikely to have low BMD with the intention of postponing DXA, a high threshold, with a good NPV, favouring sensitivity over specificity may be appropriate. CT-HU of 170–190 yielded sensitivities of 87–90%, and NPV 71–75%. Postponement of DXA and anti-resorptives would be reasonable at those levels. Conversely, if intending to initiate pharmacologic treatment based on CT densitometry, then a threshold that favours specificity will reduce false positives and unnecessary pharmacotherapy. CT-HU of 90–110 yielded specificities of 85–93%. At those extremely low levels, urgent BMD interventions should be initiated before or at the commencement of cancer treatments that will compromise BMD further. The combination of high specificity and relatively low positive predictive values shown at the lowest CT-HU thresholds are considered to potentially be due to DXA false negatives. Our evolving recommendations are FRAX assessment, calcium and vitamin D assay, consideration of vitamin D and calcium supplementation and based on CT-HU the commencement of antiresorptive medications and urgent DXA for those at high risk. We expect that the baseline DXA may be supplanted by opportunistic CT-HU assessment in gynecological oncology because a great majority of patients undergo staging CT scans.

CT may be regarded as more accurate than DXA [24,25]. DXA is a projectional technique, confounded by degenerative change, vascular calcification, osteophytes and spinal deformities, resulting in an overestimation of BMD [24]. Fifty percent of patients with moderate/severe radiographic vertebral fractures have non-osteoporotic DXA T-scores [19]. The American College of Radiology considers CT superior to DXA in patients with extensive degenerative changes or obesity [25]. CT has better sensitivity to BMD alterations caused by bone modifying agents and disease progression due to its specific assessment of trabecular bone only [25,26]. One of our cohorts had a diagnosis of concomitant vertebral fractures despite DXA categorisation of osteopaenia. Graffy et al. identified 90 HU as the optimum threshold for determining prevalent vertebral fractures in a review of almost 2000 CT scans [27]. The odds ratio for concurrent moderate/severe vertebral fractures at <90 HU was 31.9, with a prevalence of 32.5%.

To date, no thresholds for BMD assessment by CT have been incorporated into clinical guidelines. Contributing factors may be a lack of an agreed standardised method of assessing BMD on CT, CT acquisition heterogeneity and a paucity of data on normative ranges at various kilovoltages. Variations in the method of assessing BMD on CT include the choice of assessment site, radiographic plane, and use of calibration. We support the choice of the lumbar spine. A large study of white women aged >65 found 16% had osteoporosis at the lumbar spine but not at the hip [28]. The L1 vertebra has been favoured in ours and other studies as it is readily identifiable, included on most CT examinations, has the fewest degenerative changes, and has the strongest vertebral correlation with T-scores [19]. We measured BMD on axial formats and performed vertebral fracture assessments on sagittal reconstructions. Most other studies have used the same planes, though some espouse the use of sagittal reconstructions only [29].

Further advances in the assessment of bone strength and fracture prediction are expected. Automated BMD assessment software would optimise efficiency, particularly if widespread screening is employed [17]. There is a strong correlation between CT-HU at different kilovoltages. The development of kilovoltage-specific predictive algorithms would substantially broaden the applicability of opportunistic CT BMD assessment [17,30].

## 5. Conclusions

We have demonstrated a significant correlation between BMD assessment in CT-HU and DXA areal BMD, T-scores, and diagnostic categories. Strong AUC results for the diagnosis of low BMD (0.77) and osteoporosis (0.80) support the use of CT in opportunistic BMD assessment in the gynecological oncology setting.

The selection of action thresholds must acknowledge the inherent heterogeneity in opportunistic assessment, as well as the limited availability of DXA, making CT-based triage of referrals useful. Routine assessment of BMD on staging CT imaging would also provide a baseline for future, highly accurate, comparative assessment if follow-up CT is required in the patient’s cancer pathway.

Further high-quality large studies are required on this topic, and we do not currently recommend that CT is applied routinely for bone mineral density assessment, but instead suggest its utility in the opportunistic estimation of bone health. We recommend a “traffic-light system” with regard to diagnosing and managing baseline BMD deficiency based on staging CT scans: a low threshold, <90 HU, to create a red-zone with a good PPV identifying those in need of urgent DXA, and commencement of pharmacotherapy; a green-zone, above a high threshold, >190 HU, with a good NPV to select women at least risk, for whom a DXA can be postponed; an intermediate orange-zone, would incorporate all those women for whom early rather than immediate DXA would suffice. Opportunistic CT assessment of bone mineral density holds the promise of supplanting DXA in the baseline assessment of bone health in women with gynecological cancer.

## Figures and Tables

**Table 1:** Cohort Demographics and Clinical Risk Factors (*N* = 50).

Age	Median (range)	57	(31–86)
BMI	Median (range)	30.4	(15.6–57.8)
		*N*	(%)
Smoking Status	Current Smokers	9	(18%)
	Ex-smokers	15	(30%)
	Never-smokers	26	(52%)
Menopausal Status	Premenopausal	13	(26%)
	Post-menopausal	37	(74%)
Alcohol intake	<10 units/week	32	(64%)
	10–20 units/week	2	(4%)
	>20 units/week	3	(6%)
Tumour Site	Endometrium	29	(58%)
	Tubal/ovarian/peritoneal	7	(14%)
	Cervix	11	(22%)
	Vulva	2	(4%)
	Unknown	1	(2%)
Treatment	Bilateral Oophorectomy	40	(80%)
	Ext. Beam Radiotherapy	21	(42%)
	Chemotherapy	18	(36%)

**Table 2:** Mean T-score, mean areal BMD, and mean CT-HU for women in each DXA derived diagnostic category.

DXA Categories	T-Score (Mean)	Areal BMD (Mean)	CT-HU (Mean)
Normal (*n* = 17)	−0.3 SD	1.3 g/cm^2^	174 HU (95%CI 154–193)
Osteopaenia (*n* = 26)	−1.7 SD	1.1 g/cm^2^	134 HU (95%CI 112–155)
Osteoporosis (*n* = 7)	−2.8 SD	1.0 g/cm^2^	112 HU (95%CI 98–126)

**Table 3:** Areas under the curve for diagnosis of low and osteoporotic bone mineral density by CT attenuation at each vertebral level.

Vertebral Level	AUC	St. Error	95% CI	Significance
T12 Low BMD	0.754	0.078	0.60–0.91	0.004
Osteoporosis	0.698	0.079	0.54–0.85	0.119
L1 Low BMD	0.771	0.075	0.62–0.92	0.002
Osteoporosis	0.796	0.064	0.67–0.92	0.020
L2 Low BMD	0.789	0.073	0.65–0.93	0.001
Osteoporosis	0.758	0.079	0.60–0.91	0.043
L3 Low BMD	0.786	0.074	0.64–0.93	0.001
Osteoporosis	0.702	0.077	0.55–0.85	0.112
L4 Low BMD	0.811	0.065	0.68–0.94	0.000
Osteoporosis	0.802	0.066	0.67–0.93	0.018
L5 Low BMD	0.759	0.075	0.61–0.91	0.003
Osteoporosis	0.738	0.068	0.61–0.87	0.061
Avg. Low BMD	0.792	0.071	0.65–0.93	0.001
Osteoporosis	0.750	0.070	0.61–0.89	0.050

Significance at *p* < 0.05.

**Table 4:** Clinically useful thresholds for identifying low BMD and osteoporosis, with Youdon’s J-statistic thresholds for L1.

**Threshold above which patients are less likely to have low BMD**				
**CT-HU**	**Sensitivity (%)**	**Specificity (%)**	**PPV (%)**	**NPV (%)**
170	87.1	70.59	84.38	75
180	87.1	58.82	79.41	71.43
190	90.3	47.1	75.7	72.7
200	90.3	23.5	68.3	57.1
**Threshold below which patients are more likely to have osteoporosis/vertebral fracture**				
**CT-HU**	**Sensitivity (%)**	**Specificity (%)**	**PPV (%)**	**NPV (%)**
90	14.3	92.7	25	86.4
100	14.3	90.24	20	86.1
110	42.9	85.4	33.3	89.74
120	85.7	75.6	37.5	96.9
**Youdon Index; statistically optimal CT-HU diagnostic thresholds for L1**				
	**CT-HU**	**Sensitivity (%)**	**Specificity (%)**	**Youden’s J stat.**
**Low BMD**	168	87	70	0.577
**Osteoporosis**	134	100	64	0.643

## Data Availability

All relevant data available on request.

## Supplementary Materials

The following are available online at https://www.mdpi.com/article/10.3390/medicina57121386/s1, Table Legends for Supplementary Material. Table S1. A complete record of all thresholds with diagnostic performance for low bone mineral density at each vertebral level. Table S2. A complete record of all thresholds with diagnostic performance for osteoporosis at each vertebral level.

## Appendix A

The mean areal BMD and CT-HU for each vertebral level is summarised in Table A1.

**Table A1:** Mean Areal BMD and mean CT-HU for each vertebral level.

Lumbar Levels	Areal BMD (Mean)	CT-HU (Mean)
T12	-	153 HU
L1	1.07 g/cm^2^	149 HU
L2	1.15 g/cm^2^	145 HU
L3	1.19 g/cm^2^	139 HU
L4	1.22 g/cm^2^	141 HU
L5	-	145 HU

A One-way ANOVA was performed to assess the significance of the variance in mean CT-HU for each of the DXA derived diagnostic categories, at each vertebral level. A statistically significant difference was demonstrated between the mean CT-HU in each DXA diagnostic category, for each of the vertebral levels. The results of the ANOVA and post-hoc analyses are shown in Table A2.

**Table A2:** One-way ANOVA to assess the significance of the variance in mean CT-HU for each of the DXA derived diagnostic categories, at each vertebral level.

Vertebral Level	ANOVA (*p* Value)	Normal—Osteopaenia (*p* Value)	Normal—Osteoporosis (*p* Value)	Osteopaenia-Osteoporosis (*p* Value)
T12	0.02 *			
Hochberg GT2		0.049 *	0.059	0.809
Games-Howell		0.046 *	0.001 *	0.399
Dunnett t		0.016 *	0.019 *	N/A
L1	0.008 *			
Hochberg GT2		0.033 *	0.02 *	0.59
Games-Howell		0.032 *	0.0 *	0.11
Dunnett t		0.011 *	0.007 *	N/A
L2	0.004 *			
Hochberg GT2		0.016 *	0.012 *	0.574
Games-Howell		0.013 *	0.0028	0.285
Dunnett t		0.005 *	0.004 *	N/A
L3	0.006 *			
Hochberg GT2		0.015 *	0.03 *	0.826
Games-Howell		0.015 *	0.0 *	0.44
Dunnett t		0.005 *	0.01 *	N/A
L4	0.002 *			
Hochberg GT2		0.009 *	0.006 *	0.488
Games-Howell		0.007 *	0.0 *	0.129
Dunnett t		0.003 *	0.002 *	N/A
L5	0.008 *			
Hochberg GT2		0.033 *	0.022 *	0.615
Games-Howell		0.029 *	0.0 *	0.154
Dunnett t		0.011 *	0.007*	N/A
Average	0.005 *			
Hochberg GT2		0.018 *	0.015 *	0.631
Games-Howell		0.016 *	0.0 *	0.17
Dunnett t		0.006 *	0.005 *	N/A

* Significance at *p ≤* 0.05.

The diagnostic performance of assessment at the L1 vertebral level for diagnosing low BMD is shown in Table A3 with a complete record of cut-offs and their sensitivities and specificities positive and negative predictive values.

**Table A3:** Diagnostic performance at L1—low BMD (Osteopaenia and Osteoporosis).

Threshold (HU)	SENS	SPEC	PPV	NPV	ACCURACY
90	12.9	100	100	38.64	43.75
100	16.13	100	100	39.53	45.83
110	22.58	88.24	77.78	38.46	45.83
120	41.94	82.35	81.25	43.75	56.25
130	54.84	82.35	85	50	64.58
140	67.74	82.35	87.5	58.33	72.92
150	74.19	76.47	85.19	61.9	75
160	80.65	70.59	83.33	66.67	77.08
170	87.1	70.59	84.38	75	81.25
180	87.1	58.82	79.41	71.43	77.08
190	90.3	47.1	75.7	72.7	75
200	90.3	23.5	68.3	57.1	66

The diagnostic performance of assessment at the L1 vertebral level for diagnosing osteoporosis is shown in Table A4 with a complete record of cut-offs and their sensitivities and specificities positive and negative predictive values.

**Table A4:** Diagnostic performance at L1-osteoporosis/vertebral fractures (DXA T-score ≤ −2.5).

Threshold (HU)	SENS	SPEC	PPV	NPV	ACCURACY
90	14.3	92.7	25	86.4	81.25
100	14.3	90.24	20	86.1	79.2
110	42.9	85.4	33.3	89.74	79.2
120	85.7	75.6	37.5	96.9	77.08
130	85.7	65.9	30	96.4	68.8
140	100	58.5	29.2	100	64.6
150	100	51.2	25.9	100	58.33
160	100	43.9	23.33	100	52.1
170	100	39	21.9	100	47.9
180	100	34	20.6	100	43.8
