# Supplementary material for: Accuracy of Opportunistic Bone Mineral Density Assessment on Staging Computed Tomography for Gynaecological Cancers
Source: Medicina (Kaunas). 2021 Dec 20;57(12):1386. doi: 10.3390/medicina57121386 (PMC8703431; doi:10.3390/medicina57121386)
Supplement: Supplementary file 1 [file medicina-57-01386-s001.zip › medicina-1487031-supplementary.pdf]

Table S1: Threshold analysis to identify the statistically optimal CT-HU cut-off level for diagnosis of low BMD.

| **T12** | Value equal or lower than | Sensitivity | 1-Specificity | Specificity | Youden J stat |
|---|---|---|---|---|---|
| | 42.0 | 0.000 | 0.000 | 1.000 | 0.000 |
| | 64.0 | .032 | 0.000 | 1.000 | 0.032 |
| | 93.5 | .097 | 0.000 | 1.000 | 0.097 |
| | 103.0 | .129 | .059 | 0.941 | 0.070 |
| | 106.5 | .194 | .059 | 0.941 | 0.135 |
| | 109.5 | .194 | .118 | 0.882 | 0.076 |
| | 110.5 | .226 | .118 | 0.882 | 0.108 |
| | 114.0 | .258 | .118 | 0.882 | 0.140 |
| | 118.0 | .290 | .118 | 0.882 | 0.173 |
| | 120.0 | .355 | .118 | 0.882 | 0.237 |
| | 122.5 | .387 | .118 | 0.882 | 0.269 |
| | 125.0 | .387 | .176 | 0.824 | 0.211 |
| | 126.5 | .419 | .176 | 0.824 | 0.243 |
| | 129.5 | .484 | .176 | 0.824 | 0.307 |
| | 132.5 | .516 | .176 | 0.824 | 0.340 |
| | 133.5 | .548 | .176 | 0.824 | 0.372 |
| | 138.0 | .581 | .176 | 0.824 | 0.404 |
| | 142.5 | .613 | .176 | 0.824 | 0.436 |
| | 144.0 | .613 | .235 | 0.765 | 0.378 |
| | 146.5 | .645 | .235 | 0.765 | 0.410 |
| | 149.0 | .677 | .235 | 0.765 | 0.442 |
| | 152.0 | .710 | .235 | 0.765 | 0.474 |
| | 156.0 | .742 | .235 | 0.765 | 0.507 |
| | 159.0 | .774 | .235 | 0.765 | 0.539 |
| | 163.0 | .774 | .294 | 0.706 | 0.480 |
| | 167.0 | .806 | .294 | 0.706 | 0.512 |
| | 168.5 | .806 | .353 | 0.647 | 0.454 |
| | 171.5 | .839 | .412 | 0.588 | 0.427 |
| | 176.0 | .839 | .471 | 0.529 | 0.368 |
| | 179.5 | .871 | .471 | 0.529 | 0.400 |
| | 183.5 | .903 | .529 | 0.471 | 0.374 |
| | 194.5 | .903 | .588 | 0.412 | 0.315 |
| | 204.0 | .903 | .706 | 0.294 | 0.197 |
| | 207.5 | .935 | .706 | 0.294 | 0.230 |
| | 214.0 | .935 | .824 | 0.176 | 0.112 |
| | 222.0 | .935 | .882 | 0.118 | 0.053 |
| | 232.0 | .935 | .941 | 0.059 | -0.006 |
| | 246.5 | .935 | 1.000 | 0.000 | -0.065 |
| | 267.5 | .968 | 1.000 | 0.000 | -0.032 |
| | 281.0 | 1.000 | 1.000 | 0.000 | 0.000 |

Table S1: Threshold analysis to identify the statistically optimal CT-HU cut-off level for diagnosis of low BMD.

| **L1** | Value equal or lower than | Sensitivity | 1 - Specificity | Specificity | Youden J stat |
|---|---|---|---|---|---|
| | -1 | 0 | 0 | 1 | 0 |
| | 41 | 0.032 | 0 | 1 | 0.032 |
| | 84 | 0.065 | 0 | 1 | 0.065 |
| | 88 | 0.097 | 0 | 1 | 0.097 |
| | 91 | 0.129 | 0 | 1 | 0.129 |
| | 97.5 | 0.161 | 0 | 1 | 0.161 |
| | 104 | 0.194 | 0 | 1 | 0.194 |
| | 105.5 | 0.226 | 0 | 1 | 0.226 |
| | 108.5 | 0.226 | 0.118 | 0.882 | 0.108 |
| | 111.5 | 0.29 | 0.118 | 0.882 | 0.172 |
| | 113 | 0.323 | 0.118 | 0.882 | 0.205 |
| | 115.5 | 0.355 | 0.176 | 0.824 | 0.179 |
| | 118.5 | 0.387 | 0.176 | 0.824 | 0.211 |
| | 121 | 0.419 | 0.176 | 0.824 | 0.243 |
| | 124.5 | 0.452 | 0.176 | 0.824 | 0.276 |
| | 128 | 0.484 | 0.176 | 0.824 | 0.308 |
| | 129.5 | 0.516 | 0.176 | 0.824 | 0.34 |
| | 130.5 | 0.548 | 0.176 | 0.824 | 0.372 |
| | 134 | 0.581 | 0.176 | 0.824 | 0.405 |
| | 139.5 | 0.677 | 0.176 | 0.824 | 0.501 |
| | 142.5 | 0.71 | 0.176 | 0.824 | 0.534 |
| | 146.5 | 0.71 | 0.235 | 0.765 | 0.475 |
| | 150.5 | 0.742 | 0.235 | 0.765 | 0.507 |
| | 154 | 0.774 | 0.235 | 0.765 | 0.539 |
| | 157.5 | 0.774 | 0.294 | 0.706 | 0.48 |
| | 159.5 | 0.806 | 0.294 | 0.706 | 0.512 |
| | 162 | 0.839 | 0.294 | 0.706 | 0.545 |
| | 168 | 0.871 | 0.294 | 0.706 | 0.577 |
| | 175 | 0.871 | 0.353 | 0.647 | 0.518 |
| | 178.5 | 0.871 | 0.412 | 0.588 | 0.459 |
| | 180.5 | 0.871 | 0.471 | 0.529 | 0.4 |
| | 184.5 | 0.871 | 0.529 | 0.471 | 0.342 |
| | 190.5 | 0.903 | 0.529 | 0.471 | 0.374 |
| | 193.5 | 0.903 | 0.588 | 0.412 | 0.315 |
| | 196.5 | 0.903 | 0.706 | 0.294 | 0.197 |
| | 200 | 0.903 | 0.765 | 0.235 | 0.138 |
| | 203.5 | 0.903 | 0.824 | 0.176 | 0.079 |
| | 207 | 0.935 | 0.824 | 0.176 | 0.111 |
| | 213.5 | 0.935 | 0.882 | 0.118 | 0.053 |
| | 232.5 | 0.935 | 0.941 | 0.059 | -0.006 |
| | 255.5 | 0.968 | 0.941 | 0.059 | 0.027 |
| | 269.5 | 0.968 | 1 | 0 | -0.032 |
| | 275 | 1 | 1 | 0 | 0 |

Table S1: Threshold analysis to identify the statistically optimal CT-HU cut-off level for diagnosis of low BMD.

| **L2** | Value equal or lower than | Sensitivity | 1 - Specificity | Specificity | Youden J stat |
|---|---|---|---|---|---|
| | 29 | 0 | 0 | 1 | 0 |
| | 50.5 | 0.032 | 0 | 1 | 0.032 |
| | 72 | 0.065 | 0 | 1 | 0.065 |
| | 76.5 | 0.097 | 0 | 1 | 0.097 |
| | 87.5 | 0.161 | 0 | 1 | 0.161 |
| | 96 | 0.194 | 0 | 1 | 0.194 |
| | 97.5 | 0.194 | 0.118 | 0.882 | 0.076 |
| | 101 | 0.226 | 0.118 | 0.882 | 0.108 |
| | 106 | 0.258 | 0.118 | 0.882 | 0.14 |
| | 111 | 0.323 | 0.118 | 0.882 | 0.205 |
| | 116.5 | 0.387 | 0.118 | 0.882 | 0.269 |
| | 119.5 | 0.452 | 0.118 | 0.882 | 0.334 |
| | 120.5 | 0.516 | 0.118 | 0.882 | 0.398 |
| | 121.5 | 0.581 | 0.118 | 0.882 | 0.463 |
| | 123.5 | 0.613 | 0.118 | 0.882 | 0.495 |
| | 127.5 | 0.645 | 0.118 | 0.882 | 0.527 |
| | 131 | 0.645 | 0.176 | 0.824 | 0.469 |
| | 135 | 0.677 | 0.176 | 0.824 | 0.501 |
| | 144 | 0.71 | 0.176 | 0.824 | 0.534 |
| | 152 | 0.774 | 0.176 | 0.824 | 0.598 |
| | 156.5 | 0.774 | 0.235 | 0.765 | 0.539 |
| | 159.5 | 0.806 | 0.235 | 0.765 | 0.571 |
| | 163 | 0.806 | 0.294 | 0.706 | 0.512 |
| | 168 | 0.839 | 0.294 | 0.706 | 0.545 |
| | 171.5 | 0.839 | 0.353 | 0.647 | 0.486 |
| | 177.5 | 0.839 | 0.412 | 0.588 | 0.427 |
| | 183.5 | 0.839 | 0.471 | 0.529 | 0.368 |
| | 186 | 0.871 | 0.471 | 0.529 | 0.4 |
| | 187.5 | 0.903 | 0.471 | 0.529 | 0.432 |
| | 189 | 0.903 | 0.529 | 0.471 | 0.374 |
| | 191 | 0.903 | 0.588 | 0.412 | 0.315 |
| | 192.5 | 0.903 | 0.647 | 0.353 | 0.256 |
| | 193.5 | 0.903 | 0.706 | 0.294 | 0.197 |
| | 195.5 | 0.935 | 0.706 | 0.294 | 0.229 |
| | 200 | 0.935 | 0.765 | 0.235 | 0.17 |
| | 206 | 0.935 | 0.824 | 0.176 | 0.111 |
| | 210 | 0.935 | 0.882 | 0.118 | 0.053 |
| | 211.5 | 0.935 | 0.941 | 0.059 | -0.006 |
| | 228 | 0.935 | 1 | 0 | -0.065 |
| | 249.5 | 0.968 | 1 | 0 | -0.032 |
| | 256 | 1 | 1 | 0 | 0 |

Table S1: Threshold analysis to identify the statistically optimal CT-HU cut-off level for diagnosis of low BMD.

| **L3** | Value equal or lower than | Sensitivity | 1-Specificity | Specificity | Youden J stat |
|---|---|---|---|---|---|
| | 29 | 0 | 0 | 1 | 0 |
| | 46.5 | 0.032 | 0 | 1 | 0.032 |
| | 71.5 | 0.097 | 0 | 1 | 0.097 |
| | 81.5 | 0.129 | 0 | 1 | 0.129 |
| | 83.5 | 0.161 | 0 | 1 | 0.161 |
| | 85.5 | 0.194 | 0 | 1 | 0.194 |
| | 87.5 | 0.194 | 0.059 | 0.941 | 0.135 |
| | 89.5 | 0.226 | 0.059 | 0.941 | 0.167 |
| | 94.5 | 0.226 | 0.118 | 0.882 | 0.108 |
| | 100.5 | 0.258 | 0.118 | 0.882 | 0.14 |
| | 105 | 0.29 | 0.118 | 0.882 | 0.172 |
| | 109 | 0.323 | 0.118 | 0.882 | 0.205 |
| | 111.5 | 0.387 | 0.118 | 0.882 | 0.269 |
| | 112.5 | 0.419 | 0.118 | 0.882 | 0.301 |
| | 114 | 0.484 | 0.118 | 0.882 | 0.366 |
| | 115.5 | 0.516 | 0.118 | 0.882 | 0.398 |
| | 116.5 | 0.516 | 0.176 | 0.824 | 0.34 |
| | 120 | 0.548 | 0.176 | 0.824 | 0.372 |
| | 123.5 | 0.581 | 0.176 | 0.824 | 0.405 |
| | 124.5 | 0.613 | 0.176 | 0.824 | 0.437 |
| | 129.5 | 0.645 | 0.176 | 0.824 | 0.469 |
| | 134.5 | 0.677 | 0.176 | 0.824 | 0.501 |
| | 136.5 | 0.71 | 0.176 | 0.824 | 0.534 |
| | 140 | 0.742 | 0.176 | 0.824 | 0.566 |
| | 144.5 | 0.774 | 0.176 | 0.824 | 0.598 |
| | 148.5 | 0.839 | 0.176 | 0.824 | 0.663 |
| | 151 | 0.839 | 0.235 | 0.765 | 0.604 |
| | 153 | 0.839 | 0.294 | 0.706 | 0.545 |
| | 157 | 0.839 | 0.353 | 0.647 | 0.486 |
| | 160.5 | 0.839 | 0.412 | 0.588 | 0.427 |
| | 162 | 0.871 | 0.412 | 0.588 | 0.459 |
| | 165 | 0.871 | 0.471 | 0.529 | 0.4 |
| | 172.5 | 0.871 | 0.529 | 0.471 | 0.342 |
| | 183 | 0.903 | 0.529 | 0.471 | 0.374 |
| | 189.5 | 0.903 | 0.588 | 0.412 | 0.315 |
| | 191.5 | 0.903 | 0.647 | 0.353 | 0.256 |
| | 192.5 | 0.903 | 0.706 | 0.294 | 0.197 |
| | 193.5 | 0.903 | 0.765 | 0.235 | 0.138 |
| | 195 | 0.903 | 0.824 | 0.176 | 0.079 |
| | 198 | 0.935 | 0.824 | 0.176 | 0.111 |
| | 207 | 0.935 | 0.882 | 0.118 | 0.053 |
| | 216 | 0.935 | 0.941 | 0.059 | -0.006 |
| | 225 | 0.935 | 1 | 0 | -0.065 |
| | 238 | 0.968 | 1 | 0 | -0.032 |
| | 245 | 1 | 1 | 0 | 0 |

Table S1: Threshold analysis to identify the statistically optimal CT-HU cut-off level for diagnosis of low BMD.

| **L4** | Value equal or lower than | Sensitivity | 1-Specificity | Specificity | Youden J stat |
|---|---|---|---|---|---|
| | 2 | 0 | 0 | 1 | 0 |
| | 36 | 0.032 | 0 | 1 | 0.032 |
| | 69.5 | 0.065 | 0 | 1 | 0.065 |
| | 71 | 0.097 | 0 | 1 | 0.097 |
| | 72.5 | 0.129 | 0 | 1 | 0.129 |
| | 77.5 | 0.161 | 0 | 1 | 0.161 |
| | 82.5 | 0.194 | 0 | 1 | 0.194 |
| | 88 | 0.226 | 0 | 1 | 0.226 |
| | 94.5 | 0.258 | 0 | 1 | 0.258 |
| | 96.5 | 0.323 | 0 | 1 | 0.323 |
| | 100.5 | 0.323 | 0.059 | 0.941 | 0.264 |
| | 104.5 | 0.355 | 0.059 | 0.941 | 0.296 |
| | 107.5 | 0.419 | 0.059 | 0.941 | 0.36 |
| | 112.5 | 0.452 | 0.118 | 0.882 | 0.334 |
| | 116 | 0.516 | 0.118 | 0.882 | 0.398 |
| | 120 | 0.548 | 0.118 | 0.882 | 0.43 |
| | 124.5 | 0.581 | 0.118 | 0.882 | 0.463 |
| | 128 | 0.613 | 0.118 | 0.882 | 0.495 |
| | 130.5 | 0.613 | 0.176 | 0.824 | 0.437 |
| | 132.5 | 0.645 | 0.176 | 0.824 | 0.469 |
| | 135.5 | 0.677 | 0.176 | 0.824 | 0.501 |
| | 137.5 | 0.71 | 0.176 | 0.824 | 0.534 |
| | 142.5 | 0.742 | 0.176 | 0.824 | 0.566 |
| | 148.5 | 0.774 | 0.235 | 0.765 | 0.539 |
| | 152 | 0.806 | 0.235 | 0.765 | 0.571 |
| | 157 | 0.806 | 0.294 | 0.706 | 0.512 |
| | 164 | 0.806 | 0.353 | 0.647 | 0.453 |
| | 170 | 0.839 | 0.353 | 0.647 | 0.486 |
| | 173.5 | 0.903 | 0.412 | 0.588 | 0.491 |
| | 179 | 0.903 | 0.471 | 0.529 | 0.432 |
| | 184 | 0.903 | 0.529 | 0.471 | 0.374 |
| | 187 | 0.903 | 0.588 | 0.412 | 0.315 |
| | 194.5 | 0.903 | 0.647 | 0.353 | 0.256 |
| | 201 | 0.903 | 0.706 | 0.294 | 0.197 |
| | 204.5 | 0.935 | 0.765 | 0.235 | 0.17 |
| | 210 | 0.935 | 0.824 | 0.176 | 0.111 |
| | 215 | 0.935 | 0.882 | 0.118 | 0.053 |
| | 222.5 | 0.935 | 0.941 | 0.059 | -0.006 |
| | 233 | 0.935 | 1 | 0 | -0.065 |
| | 252 | 0.968 | 1 | 0 | -0.032 |
| | 267 | 1 | 1 | 0 | 0 |

Table S1: Threshold analysis to identify the statistically optimal CT-HU cut-off level for diagnosis of low BMD.

| **L5** | Value equal or lower than | Sensitivity | 1-Specificity | Specificity | Youden J Stat |
|---|---|---|---|---|---|
| | 22 | 0 | 0 | 1 | 0 |
| | 46 | 0.032 | 0 | 1 | 0.032 |
| | 71 | 0.065 | 0 | 1 | 0.065 |
| | 73.5 | 0.065 | 0.059 | 0.941 | 0.006 |
| | 78 | 0.097 | 0.059 | 0.941 | 0.038 |
| | 83 | 0.129 | 0.059 | 0.941 | 0.07 |
| | 87.5 | 0.161 | 0.059 | 0.941 | 0.102 |
| | 93 | 0.194 | 0.059 | 0.941 | 0.135 |
| | 97 | 0.226 | 0.059 | 0.941 | 0.167 |
| | 100 | 0.258 | 0.059 | 0.941 | 0.199 |
| | 102 | 0.29 | 0.059 | 0.941 | 0.231 |
| | 103.5 | 0.323 | 0.059 | 0.941 | 0.264 |
| | 104.5 | 0.323 | 0.118 | 0.882 | 0.205 |
| | 105.5 | 0.355 | 0.118 | 0.882 | 0.237 |
| | 107 | 0.387 | 0.118 | 0.882 | 0.269 |
| | 109 | 0.419 | 0.118 | 0.882 | 0.301 |
| | 111.5 | 0.484 | 0.118 | 0.882 | 0.366 |
| | 116 | 0.516 | 0.118 | 0.882 | 0.398 |
| | 121 | 0.548 | 0.118 | 0.882 | 0.43 |
| | 127.5 | 0.581 | 0.118 | 0.882 | 0.463 |
| | 133 | 0.613 | 0.118 | 0.882 | 0.495 |
| | 136.5 | 0.645 | 0.118 | 0.882 | 0.527 |
| | 140.5 | 0.645 | 0.176 | 0.824 | 0.469 |
| | 142.5 | 0.677 | 0.176 | 0.824 | 0.501 |
| | 144 | 0.71 | 0.176 | 0.824 | 0.534 |
| | 150 | 0.742 | 0.176 | 0.824 | 0.566 |
| | 157 | 0.774 | 0.176 | 0.824 | 0.598 |
| | 160 | 0.774 | 0.235 | 0.765 | 0.539 |
| | 162 | 0.774 | 0.294 | 0.706 | 0.48 |
| | 164 | 0.774 | 0.353 | 0.647 | 0.421 |
| | 167.5 | 0.774 | 0.412 | 0.588 | 0.362 |
| | 171 | 0.806 | 0.412 | 0.588 | 0.394 |
| | 175 | 0.806 | 0.471 | 0.529 | 0.335 |
| | 178.5 | 0.806 | 0.529 | 0.471 | 0.277 |
| | 180.5 | 0.839 | 0.529 | 0.471 | 0.31 |
| | 183 | 0.839 | 0.588 | 0.412 | 0.251 |
| | 188.5 | 0.839 | 0.647 | 0.353 | 0.192 |
| | 197 | 0.871 | 0.647 | 0.353 | 0.224 |
| | 202 | 0.903 | 0.647 | 0.353 | 0.256 |
| | 203.5 | 0.903 | 0.706 | 0.294 | 0.197 |
| | 206 | 0.903 | 0.765 | 0.235 | 0.138 |
| | 208.5 | 0.903 | 0.824 | 0.176 | 0.079 |
| | 211 | 0.903 | 0.882 | 0.118 | 0.021 |
| | 214 | 0.935 | 0.882 | 0.118 | 0.053 |
| | 218 | 0.935 | 0.941 | 0.059 | -0.006 |
| | 222.5 | 0.935 | 1 | 0 | -0.065 |
| | 242 | 0.968 | 1 | 0 | -0.032 |
| | 261 | 1 | 1 | 0 | 0 |

Table S1: Threshold analysis to identify the statistically optimal CT-HU cut-off level for diagnosis of low BMD.

| **Avg** | Value equal or lower than | Sensitivity | 1-Specificity | Specificity | Youden J stat |
|---|---|---|---|---|---|
| | 21 | 0 | 0 | 1 | 0 |
| | 49 | 0.032 | 0 | 1 | 0.032 |
| | 78 | 0.065 | 0 | 1 | 0.065 |
| | 82 | 0.097 | 0 | 1 | 0.097 |
| | 87 | 0.129 | 0 | 1 | 0.129 |
| | 91 | 0.161 | 0 | 1 | 0.161 |
| | 93 | 0.194 | 0 | 1 | 0.194 |
| | 96 | 0.194 | 0.059 | 0.941 | 0.135 |
| | 100 | 0.226 | 0.059 | 0.941 | 0.167 |
| | 102.5 | 0.258 | 0.059 | 0.941 | 0.199 |
| | 106.5 | 0.258 | 0.118 | 0.882 | 0.14 |
| | 111 | 0.29 | 0.118 | 0.882 | 0.172 |
| | 112.5 | 0.323 | 0.118 | 0.882 | 0.205 |
| | 115 | 0.355 | 0.118 | 0.882 | 0.237 |
| | 118 | 0.419 | 0.118 | 0.882 | 0.301 |
| | 120 | 0.452 | 0.118 | 0.882 | 0.334 |
| | 121.5 | 0.516 | 0.118 | 0.882 | 0.398 |
| | 122.5 | 0.548 | 0.118 | 0.882 | 0.43 |
| | 124 | 0.581 | 0.118 | 0.882 | 0.463 |
| | 125.5 | 0.613 | 0.118 | 0.882 | 0.495 |
| | 126.5 | 0.613 | 0.176 | 0.824 | 0.437 |
| | 129 | 0.645 | 0.176 | 0.824 | 0.469 |
| | 137.5 | 0.677 | 0.176 | 0.824 | 0.501 |
| | 145.5 | 0.71 | 0.176 | 0.824 | 0.534 |
| | 149 | 0.742 | 0.176 | 0.824 | 0.566 |
| | 154 | 0.774 | 0.176 | 0.824 | 0.598 |
| | 159.5 | 0.806 | 0.235 | 0.765 | 0.571 |
| | 162.5 | 0.806 | 0.294 | 0.706 | 0.512 |
| | 164.5 | 0.839 | 0.294 | 0.706 | 0.545 |
| | 167 | 0.839 | 0.353 | 0.647 | 0.486 |
| | 169.5 | 0.839 | 0.412 | 0.588 | 0.427 |
| | 172 | 0.839 | 0.471 | 0.529 | 0.368 |
| | 174 | 0.871 | 0.471 | 0.529 | 0.4 |
| | 180.5 | 0.871 | 0.529 | 0.471 | 0.342 |
| | 187.75 | 0.871 | 0.588 | 0.412 | 0.283 |
| | 189.75 | 0.903 | 0.588 | 0.412 | 0.315 |
| | 192 | 0.935 | 0.588 | 0.412 | 0.347 |
| | 196 | 0.935 | 0.647 | 0.353 | 0.288 |
| | 200 | 0.935 | 0.706 | 0.294 | 0.229 |
| | 203 | 0.935 | 0.765 | 0.235 | 0.17 |
| | 204.5 | 0.935 | 0.824 | 0.176 | 0.111 |
| | 211 | 0.935 | 0.882 | 0.118 | 0.053 |
| | 222.5 | 0.935 | 0.941 | 0.059 | -0.006 |
| | 234 | 0.935 | 1 | 0 | -0.065 |
| | 251.5 | 0.968 | 1 | 0 | -0.032 |
| | 264 | 1 | 1 | 0 | 0 |

Table S1: Threshold analysis to identify the statistically optimal CT-HU cut-off level for diagnosis of low BMD.

| **T12** | Value equal or lower than | Sensitivity | 1-Specificity | Specificity | Youden J stat |
|---|---|---|---|---|---|
| | 42 | 0 | 0 | 1 | 0 |
| | 64 | 0 | 0.024 | 0.976 | -0.024 |
| | 93.5 | 0 | 0.071 | 0.929 | -0.071 |
| | 103 | 0.167 | 0.095 | 0.905 | 0.072 |
| | 106.5 | 0.167 | 0.143 | 0.857 | 0.024 |
| | 109.5 | 0.167 | 0.167 | 0.833 | 0 |
| | 110.5 | 0.167 | 0.19 | 0.81 | -0.023 |
| | 114 | 0.333 | 0.19 | 0.81 | 0.143 |
| | 118 | 0.333 | 0.214 | 0.786 | 0.119 |
| | 120 | 0.333 | 0.262 | 0.738 | 0.071 |
| | 122.5 | 0.333 | 0.286 | 0.714 | 0.047 |
| | 125 | 0.333 | 0.31 | 0.69 | 0.023 |
| | 126.5 | 0.333 | 0.333 | 0.667 | 0 |
| | 129.5 | 0.5 | 0.357 | 0.643 | 0.143 |
| | 132.5 | 0.667 | 0.357 | 0.643 | 0.31 |
| | 133.5 | 0.667 | 0.381 | 0.619 | 0.286 |
| | 138 | 0.667 | 0.405 | 0.595 | 0.262 |
| | 142.5 | 0.833 | 0.405 | 0.595 | 0.428 |
| | 144 | 0.833 | 0.429 | 0.571 | 0.404 |
| | 146.5 | 1 | 0.429 | 0.571 | 0.571 |
| | 149 | 1 | 0.452 | 0.548 | 0.548 |
| | 152 | 1 | 0.476 | 0.524 | 0.524 |
| | 156 | 1 | 0.5 | 0.5 | 0.5 |
| | 159 | 1 | 0.524 | 0.476 | 0.476 |
| | 163 | 1 | 0.548 | 0.452 | 0.452 |
| | 167 | 1 | 0.571 | 0.429 | 0.429 |
| | 168.5 | 1 | 0.595 | 0.405 | 0.405 |
| | 171.5 | 1 | 0.643 | 0.357 | 0.357 |
| | 176 | 1 | 0.667 | 0.333 | 0.333 |
| | 179.5 | 1 | 0.69 | 0.31 | 0.31 |
| | 183.5 | 1 | 0.738 | 0.262 | 0.262 |
| | 194.5 | 1 | 0.762 | 0.238 | 0.238 |
| | 204 | 1 | 0.81 | 0.19 | 0.19 |
| | 207.5 | 1 | 0.833 | 0.167 | 0.167 |
| | 214 | 1 | 0.881 | 0.119 | 0.119 |
| | 222 | 1 | 0.905 | 0.095 | 0.095 |
| | 232 | 1 | 0.929 | 0.071 | 0.071 |
| | 246.5 | 1 | 0.952 | 0.048 | 0.048 |
| | 267.5 | 1 | 0.976 | 0.024 | 0.024 |
| | 281 | 1 | 1 | 0 | 0 |

Table S2. Threshold analysis to identify the statistically optimal CT-HU cut-off level for diagnosis of Osteoporosis.

| **L1** | Value equal or lower than | Sensitivity | 1-Specificity | Specificity | Youden J stat |
|---|---|---|---|---|---|
| | -1 | 0 | 0 | 1 | 0 |
| | 41 | 0 | 0.024 | 0.976 | -0.024 |
| | 84 | 0 | 0.048 | 0.952 | -0.048 |
| | 88 | 0 | 0.071 | 0.929 | -0.071 |
| | 91 | 0 | 0.095 | 0.905 | -0.095 |
| | 97.5 | 0 | 0.119 | 0.881 | -0.119 |
| | 104 | 0.167 | 0.119 | 0.881 | 0.048 |
| | 105.5 | 0.333 | 0.119 | 0.881 | 0.214 |
| | 108.5 | 0.333 | 0.167 | 0.833 | 0.166 |
| | 111.5 | 0.5 | 0.19 | 0.81 | 0.31 |
| | 113 | 0.667 | 0.19 | 0.81 | 0.477 |
| | 115.5 | 0.667 | 0.238 | 0.762 | 0.429 |
| | 118.5 | 0.667 | 0.262 | 0.738 | 0.405 |
| | 121 | 0.833 | 0.262 | 0.738 | 0.571 |
| | 124.5 | 0.833 | 0.286 | 0.714 | 0.547 |
| | 128 | 0.833 | 0.31 | 0.69 | 0.523 |
| | 129.5 | 0.833 | 0.333 | 0.667 | 0.5 |
| | 130.5 | 0.833 | 0.357 | 0.643 | 0.476 |
| | 134 | 1 | 0.357 | 0.643 | 0.643 |
| | 139.5 | 1 | 0.429 | 0.571 | 0.571 |
| | 142.5 | 1 | 0.452 | 0.548 | 0.548 |
| | 146.5 | 1 | 0.476 | 0.524 | 0.524 |
| | 150.5 | 1 | 0.5 | 0.5 | 0.5 |
| | 154 | 1 | 0.524 | 0.476 | 0.476 |
| | 157.5 | 1 | 0.548 | 0.452 | 0.452 |
| | 159.5 | 1 | 0.571 | 0.429 | 0.429 |
| | 162 | 1 | 0.595 | 0.405 | 0.405 |
| | 168 | 1 | 0.619 | 0.381 | 0.381 |
| | 175 | 1 | 0.643 | 0.357 | 0.357 |
| | 178.5 | 1 | 0.667 | 0.333 | 0.333 |
| | 180.5 | 1 | 0.69 | 0.31 | 0.31 |
| | 184.5 | 1 | 0.714 | 0.286 | 0.286 |
| | 190.5 | 1 | 0.738 | 0.262 | 0.262 |
| | 193.5 | 1 | 0.762 | 0.238 | 0.238 |
| | 196.5 | 1 | 0.81 | 0.19 | 0.19 |
| | 200 | 1 | 0.833 | 0.167 | 0.167 |
| | 203.5 | 1 | 0.857 | 0.143 | 0.143 |
| | 207 | 1 | 0.881 | 0.119 | 0.119 |
| | 213.5 | 1 | 0.905 | 0.095 | 0.095 |
| | 232.5 | 1 | 0.929 | 0.071 | 0.071 |
| | 255.5 | 1 | 0.952 | 0.048 | 0.048 |
| | 269.5 | 1 | 0.976 | 0.024 | 0.024 |
| | 275 | 1 | 1 | 0 | 0 |

Table S2. Threshold analysis to identify the statistically optimal CT-HU cut-off level for diagnosis of Osteoporosis.

| L2 | Value equal or lower than | Sensitivity | 1-Specificity | Specificity | Youden J stat |
|---|---|---|---|---|---|
| | 29 | 0 | 0 | 1 | 0 |
| | 50.5 | 0 | 0.024 | 0.976 | -0.024 |
| | 72 | 0.167 | 0.024 | 0.976 | 0.143 |
| | 76.5 | 0.167 | 0.048 | 0.952 | 0.119 |
| | 87.5 | 0.167 | 0.095 | 0.905 | 0.072 |
| | 96 | 0.167 | 0.119 | 0.881 | 0.048 |
| | 97.5 | 0.167 | 0.167 | 0.833 | 0 |
| | 101 | 0.333 | 0.167 | 0.833 | 0.166 |
| | 106 | 0.333 | 0.19 | 0.81 | 0.143 |
| | 111 | 0.5 | 0.214 | 0.786 | 0.286 |
| | 116.5 | 0.667 | 0.238 | 0.762 | 0.429 |
| | 119.5 | 0.667 | 0.286 | 0.714 | 0.381 |
| | 120.5 | 0.667 | 0.333 | 0.667 | 0.334 |
| | 121.5 | 0.833 | 0.357 | 0.643 | 0.476 |
| | 123.5 | 0.833 | 0.381 | 0.619 | 0.452 |
| | 127.5 | 0.833 | 0.405 | 0.595 | 0.428 |
| | 131 | 0.833 | 0.429 | 0.571 | 0.404 |
| | 135 | 0.833 | 0.452 | 0.548 | 0.381 |
| | 144 | 0.833 | 0.476 | 0.524 | 0.357 |
| | 152 | 1 | 0.5 | 0.5 | 0.5 |
| | 156.5 | 1 | 0.524 | 0.476 | 0.476 |
| | 159.5 | 1 | 0.548 | 0.452 | 0.452 |
| | 163 | 1 | 0.571 | 0.429 | 0.429 |
| | 168 | 1 | 0.595 | 0.405 | 0.405 |
| | 171.5 | 1 | 0.619 | 0.381 | 0.381 |
| | 177.5 | 1 | 0.643 | 0.357 | 0.357 |
| | 183.5 | 1 | 0.667 | 0.333 | 0.333 |
| | 186 | 1 | 0.69 | 0.31 | 0.31 |
| | 187.5 | 1 | 0.714 | 0.286 | 0.286 |
| | 189 | 1 | 0.738 | 0.262 | 0.262 |
| | 191 | 1 | 0.762 | 0.238 | 0.238 |
| | 192.5 | 1 | 0.786 | 0.214 | 0.214 |
| | 193.5 | 1 | 0.81 | 0.19 | 0.19 |
| | 195.5 | 1 | 0.833 | 0.167 | 0.167 |
| | 200 | 1 | 0.857 | 0.143 | 0.143 |
| | 206 | 1 | 0.881 | 0.119 | 0.119 |
| | 210 | 1 | 0.905 | 0.095 | 0.095 |
| | 211.5 | 1 | 0.929 | 0.071 | 0.071 |
| | 228 | 1 | 0.952 | 0.048 | 0.048 |
| | 249.5 | 1 | 0.976 | 0.024 | 0.024 |
| | 256 | 1 | 1 | 0 | 0 |

Table S2. Threshold analysis to identify the statistically optimal CT-HU cut-off level for diagnosis of Osteoporosis.

| L3 | Value equal or lower than | Sensitivity | 1-Specificity | Specificity | Youden J stat |
|---|---|---|---|---|---|
| | 29 | 0 | 0 | 1 | 0 |
| | 46.5 | 0 | 0.024 | 0.976 | -0.024 |
| | 71.5 | 0 | 0.071 | 0.929 | -0.071 |
| | 81.5 | 0 | 0.095 | 0.905 | -0.095 |
| | 83.5 | 0 | 0.119 | 0.881 | -0.119 |
| | 85.5 | 0 | 0.143 | 0.857 | -0.143 |
| | 87.5 | 0 | 0.167 | 0.833 | -0.167 |
| | 89.5 | 0.167 | 0.167 | 0.833 | 0 |
| | 94.5 | 0.167 | 0.19 | 0.81 | -0.023 |
| | 100.5 | 0.333 | 0.19 | 0.81 | 0.143 |
| | 105 | 0.5 | 0.19 | 0.81 | 0.31 |
| | 109 | 0.5 | 0.214 | 0.786 | 0.286 |
| | 111.5 | 0.5 | 0.262 | 0.738 | 0.238 |
| | 112.5 | 0.5 | 0.286 | 0.714 | 0.214 |
| | 114 | 0.5 | 0.333 | 0.667 | 0.167 |
| | 115.5 | 0.5 | 0.357 | 0.643 | 0.143 |
| | 116.5 | 0.5 | 0.381 | 0.619 | 0.119 |
| | 120 | 0.667 | 0.381 | 0.619 | 0.286 |
| | 123.5 | 0.667 | 0.405 | 0.595 | 0.262 |
| | 124.5 | 0.667 | 0.429 | 0.571 | 0.238 |
| | 129.5 | 0.833 | 0.429 | 0.571 | 0.404 |
| | 134.5 | 1 | 0.429 | 0.571 | 0.571 |
| | 136.5 | 1 | 0.452 | 0.548 | 0.548 |
| | 140 | 1 | 0.476 | 0.524 | 0.524 |
| | 144.5 | 1 | 0.5 | 0.5 | 0.5 |
| | 148.5 | 1 | 0.548 | 0.452 | 0.452 |
| | 151 | 1 | 0.571 | 0.429 | 0.429 |
| | 153 | 1 | 0.595 | 0.405 | 0.405 |
| | 157 | 1 | 0.619 | 0.381 | 0.381 |
| | 160.5 | 1 | 0.643 | 0.357 | 0.357 |
| | 162 | 1 | 0.667 | 0.333 | 0.333 |
| | 165 | 1 | 0.69 | 0.31 | 0.31 |
| | 172.5 | 1 | 0.714 | 0.286 | 0.286 |
| | 183 | 1 | 0.738 | 0.262 | 0.262 |
| | 189.5 | 1 | 0.762 | 0.238 | 0.238 |
| | 191.5 | 1 | 0.786 | 0.214 | 0.214 |
| | 192.5 | 1 | 0.81 | 0.19 | 0.19 |
| | 193.5 | 1 | 0.833 | 0.167 | 0.167 |
| | 195 | 1 | 0.857 | 0.143 | 0.143 |
| | 198 | 1 | 0.881 | 0.119 | 0.119 |
| | 207 | 1 | 0.905 | 0.095 | 0.095 |
| | 216 | 1 | 0.929 | 0.071 | 0.071 |
| | 225 | 1 | 0.952 | 0.048 | 0.048 |
| | 238 | 1 | 0.976 | 0.024 | 0.024 |
| | 245 | 1 | 1 | 0 | 0 |

Table S2. Threshold analysis to identify the statistically optimal CT-HU cut-off level for diagnosis of Osteoporosis.

| L4 | Value equal or lower than | Sensitivity | 1-Specificity | Specificity | Youden J stat |
|---|---|---|---|---|---|
| | 2 | 0 | 0 | 1 | 0 |
| | 36 | 0 | 0.024 | 0.976 | -0.024 |
| | 69.5 | 0 | 0.048 | 0.952 | -0.048 |
| | 71 | 0.167 | 0.048 | 0.952 | 0.119 |
| | 72.5 | 0.167 | 0.071 | 0.929 | 0.096 |
| | 77.5 | 0.167 | 0.095 | 0.905 | 0.072 |
| | 82.5 | 0.167 | 0.119 | 0.881 | 0.048 |
| | 88 | 0.167 | 0.143 | 0.857 | 0.024 |
| | 94.5 | 0.333 | 0.143 | 0.857 | 0.19 |
| | 96.5 | 0.5 | 0.167 | 0.833 | 0.333 |
| | 100.5 | 0.5 | 0.19 | 0.81 | 0.31 |
| | 104.5 | 0.667 | 0.19 | 0.81 | 0.477 |
| | 107.5 | 0.667 | 0.238 | 0.762 | 0.429 |
| | 112.5 | 0.667 | 0.286 | 0.714 | 0.381 |
| | 116 | 0.833 | 0.31 | 0.69 | 0.523 |
| | 120 | 0.833 | 0.333 | 0.667 | 0.5 |
| | 124.5 | 0.833 | 0.357 | 0.643 | 0.476 |
| | 128 | 1 | 0.357 | 0.643 | 0.643 |
| | 130.5 | 1 | 0.381 | 0.619 | 0.619 |
| | 132.5 | 1 | 0.405 | 0.595 | 0.595 |
| | 135.5 | 1 | 0.429 | 0.571 | 0.571 |
| | 137.5 | 1 | 0.452 | 0.548 | 0.548 |
| | 142.5 | 1 | 0.476 | 0.524 | 0.524 |
| | 148.5 | 1 | 0.524 | 0.476 | 0.476 |
| | 152 | 1 | 0.548 | 0.452 | 0.452 |
| | 157 | 1 | 0.571 | 0.429 | 0.429 |
| | 164 | 1 | 0.595 | 0.405 | 0.405 |
| | 170 | 1 | 0.619 | 0.381 | 0.381 |
| | 173.5 | 1 | 0.69 | 0.31 | 0.31 |
| | 179 | 1 | 0.714 | 0.286 | 0.286 |
| | 184 | 1 | 0.738 | 0.262 | 0.262 |
| | 187 | 1 | 0.762 | 0.238 | 0.238 |
| | 194.5 | 1 | 0.786 | 0.214 | 0.214 |
| | 201 | 1 | 0.81 | 0.19 | 0.19 |
| | 204.5 | 1 | 0.857 | 0.143 | 0.143 |
| | 210 | 1 | 0.881 | 0.119 | 0.119 |
| | 215 | 1 | 0.905 | 0.095 | 0.095 |
| | 222.5 | 1 | 0.929 | 0.071 | 0.071 |
| | 233 | 1 | 0.952 | 0.048 | 0.048 |
| | 252 | 1 | 0.976 | 0.024 | 0.024 |
| | 267 | 1 | 1 | 0 | 0 |

Table S2. Threshold analysis to identify the statistically optimal CT-HU cut-off level for diagnosis of Osteoporosis.

| L5 | Value equal or lower than | Sensitivity | 1-Specificity | Specificity | Youden J stat |
|---|---|---|---|---|---|
| | 22 | 0 | 0 | 1 | 0 |
| | 46 | 0 | 0.024 | 0.976 | -0.024 |
| | 71 | 0 | 0.048 | 0.952 | -0.048 |
| | 73.5 | 0 | 0.071 | 0.929 | -0.071 |
| | 78 | 0 | 0.095 | 0.905 | -0.095 |
| | 83 | 0 | 0.119 | 0.881 | -0.119 |
| | 87.5 | 0 | 0.143 | 0.857 | -0.143 |
| | 93 | 0 | 0.167 | 0.833 | -0.167 |
| | 97 | 0.167 | 0.167 | 0.833 | 0 |
| | 100 | 0.167 | 0.19 | 0.81 | -0.023 |
| | 102 | 0.333 | 0.19 | 0.81 | 0.143 |
| | 103.5 | 0.333 | 0.214 | 0.786 | 0.119 |
| | 104.5 | 0.333 | 0.238 | 0.762 | 0.095 |
| | 105.5 | 0.333 | 0.262 | 0.738 | 0.071 |
| | 107 | 0.5 | 0.262 | 0.738 | 0.238 |
| | 109 | 0.667 | 0.262 | 0.738 | 0.405 |
| | 111.5 | 0.667 | 0.31 | 0.69 | 0.357 |
| | 116 | 0.667 | 0.333 | 0.667 | 0.334 |
| | 121 | 0.833 | 0.333 | 0.667 | 0.5 |
| | 127.5 | 0.833 | 0.357 | 0.643 | 0.476 |
| | 133 | 1 | 0.357 | 0.643 | 0.643 |
| | 136.5 | 1 | 0.381 | 0.619 | 0.619 |
| | 140.5 | 1 | 0.405 | 0.595 | 0.595 |
| | 142.5 | 1 | 0.429 | 0.571 | 0.571 |
| | 144 | 1 | 0.452 | 0.548 | 0.548 |
| | 150 | 1 | 0.476 | 0.524 | 0.524 |
| | 157 | 1 | 0.5 | 0.5 | 0.5 |
| | 160 | 1 | 0.524 | 0.476 | 0.476 |
| | 162 | 1 | 0.548 | 0.452 | 0.452 |
| | 164 | 1 | 0.571 | 0.429 | 0.429 |
| | 167.5 | 1 | 0.595 | 0.405 | 0.405 |
| | 171 | 1 | 0.619 | 0.381 | 0.381 |
| | 175 | 1 | 0.643 | 0.357 | 0.357 |
| | 178.5 | 1 | 0.667 | 0.333 | 0.333 |
| | 180.5 | 1 | 0.69 | 0.31 | 0.31 |
| | 183 | 1 | 0.714 | 0.286 | 0.286 |
| | 188.5 | 1 | 0.738 | 0.262 | 0.262 |
| | 197 | 1 | 0.762 | 0.238 | 0.238 |
| | 202 | 1 | 0.786 | 0.214 | 0.214 |
| | 203.5 | 1 | 0.81 | 0.19 | 0.19 |
| | 206 | 1 | 0.833 | 0.167 | 0.167 |
| | 208.5 | 1 | 0.857 | 0.143 | 0.143 |
| | 211 | 1 | 0.881 | 0.119 | 0.119 |
| | 214 | 1 | 0.905 | 0.095 | 0.095 |
| | 218 | 1 | 0.929 | 0.071 | 0.071 |
| | 222.5 | 1 | 0.952 | 0.048 | 0.048 |
| | 242 | 1 | 0.976 | 0.024 | 0.024 |
| | 261 | 1 | 1 | 0 | 0 |

Table S2. Threshold analysis to identify the statistically optimal CT-HU cut-off level for diagnosis of Osteoporosis.

| **Avg** | Value equal or lower than | Sensitivity | 1-Specificity | Specificity | Youden J stat |
|---|---|---|---|---|---|
| | 21 | 0 | 0 | 1 | 0 |
| | 49 | 0 | 0.024 | 0.976 | -0.024 |
| | 78 | 0 | 0.048 | 0.952 | -0.048 |
| | 82 | 0 | 0.071 | 0.929 | -0.071 |
| | 87 | 0 | 0.095 | 0.905 | -0.095 |
| | 91 | 0 | 0.119 | 0.881 | -0.119 |
| | 93 | 0.167 | 0.119 | 0.881 | 0.048 |
| | 96 | 0.167 | 0.143 | 0.857 | 0.024 |
| | 100 | 0.167 | 0.167 | 0.833 | 0 |
| | 102.5 | 0.333 | 0.167 | 0.833 | 0.166 |
| | 106.5 | 0.333 | 0.19 | 0.81 | 0.143 |
| | 111 | 0.5 | 0.19 | 0.81 | 0.31 |
| | 112.5 | 0.5 | 0.214 | 0.786 | 0.286 |
| | 115 | 0.5 | 0.238 | 0.762 | 0.262 |
| | 118 | 0.5 | 0.286 | 0.714 | 0.214 |
| | 120 | 0.5 | 0.31 | 0.69 | 0.19 |
| | 121.5 | 0.833 | 0.31 | 0.69 | 0.523 |
| | 122.5 | 0.833 | 0.333 | 0.667 | 0.5 |
| | 124 | 0.833 | 0.357 | 0.643 | 0.476 |
| | 125.5 | 0.833 | 0.381 | 0.619 | 0.452 |
| | 126.5 | 0.833 | 0.405 | 0.595 | 0.428 |
| | 129 | 1 | 0.405 | 0.595 | 0.595 |
| | 137.5 | 1 | 0.429 | 0.571 | 0.571 |
| | 145.5 | 1 | 0.452 | 0.548 | 0.548 |
| | 149 | 1 | 0.476 | 0.524 | 0.524 |
| | 154 | 1 | 0.5 | 0.5 | 0.5 |
| | 159.5 | 1 | 0.548 | 0.452 | 0.452 |
| | 162.5 | 1 | 0.571 | 0.429 | 0.429 |
| | 164.5 | 1 | 0.595 | 0.405 | 0.405 |
| | 167 | 1 | 0.619 | 0.381 | 0.381 |
| | 169.5 | 1 | 0.643 | 0.357 | 0.357 |
| | 172 | 1 | 0.667 | 0.333 | 0.333 |
| | 174 | 1 | 0.69 | 0.31 | 0.31 |
| | 180.5 | 1 | 0.714 | 0.286 | 0.286 |
| | 187.75 | 1 | 0.738 | 0.262 | 0.262 |
| | 189.75 | 1 | 0.762 | 0.238 | 0.238 |
| | 192 | 1 | 0.786 | 0.214 | 0.214 |
| | 196 | 1 | 0.81 | 0.19 | 0.19 |
| | 200 | 1 | 0.833 | 0.167 | 0.167 |
| | 203 | 1 | 0.857 | 0.143 | 0.143 |
| | 204.5 | 1 | 0.881 | 0.119 | 0.119 |
| | 211 | 1 | 0.905 | 0.095 | 0.095 |
| | 222.5 | 1 | 0.929 | 0.071 | 0.071 |
| | 234 | 1 | 0.952 | 0.048 | 0.048 |
| | 251.5 | 1 | 0.976 | 0.024 | 0.024 |
| | 264 | 1 | 1 | 0 | 0 |
